# The crosstalk between BAT thermogenesis and skeletal muscle dysfunction

**DOI:** 10.3389/fphys.2023.1132830

**Published:** 2023-04-21

**Authors:** Yao Chen, Qian Hu, Changyi Wang, Tiantian Wang

**Affiliations:** ^1^ Department of Breast Surgery, West China Hospital, Sichuan University, Chengdu, China; ^2^ Health Management Center, West China Hospital of Sichuan University, Chengdu, Sichuan, China; ^3^ Department of Rehabilitation Medicine, Key Laboratory of Rehabilitation Medicine, West China Hospital, Sichuan University, Chengdu, Sichuan, China; ^4^ Institute of Rehabilitation Medicine, West China Hospital, Sichuan University, Chengdu, Sichuan, China

**Keywords:** metabolic defects, thermogenesis, brown adipose tissue, insulin resistance, skeletal muscle

## Abstract

Metabolic defects increase the risk of skeletal muscle diseases, and muscle impairment might worsen metabolic disruption, leading to a vicious cycle. Both brown adipose tissue (BAT) and skeletal muscle play important roles in non-shivering thermogenesis to regulate energy homeostasis. BAT regulates body temperature, systemic metabolism, and seretion of batokines that have positive or negative impacts on skeletal muscle. Conversely, muscle can secrete myokines that regulate BAT function. This review explained the crosstalk between BAT and skeletal muscle, and then discussed the batokines and highlighted their impact on skeletal muscle under physiological conditions. BAT is now considered a potential therapeutic target for obesity and diabetes treatment. Moreover, manipulation of BAT may be an attractive approach for the treatment of muscle weakness by correcting metabolic deficits. Therefore, exploring BAT as a potential treatment for sarcopenia could be a promising avenue for future research.

## 1 Introduction

Adipose tissue is a vital organ that plays a crutial role in energy metabolism, insulin sensitivity, and energy balance of the body. It consists of two main types: white fat tissue (WAT) and brown fat tissue (BAT). Additionally, skeletal muscle is an endocrine organ that can influence other tissues in the body. While numerous studies have explored the functions of BAT and skeletal muscle in maintaining energy homeostasis, the exact mechanism of the direct crosstalk between BAT and muscle dysfunction remains unclear (Bal et al., 2017).

The interscapular depot may be a prominent site of BAT in rodents, playing a crucial role in systemic energyhomeostasis through thermogenesis (Betz and Enerback, 2018). Brown and beige adipocytes are some of the major sites to catabolize stored energy to generate heat by non-shivering thermogenesis (NST) through uncoupling protein-1 (UCP-1) (Ikeda and Yamada, 2020; Roesler and Kazak, 2020). By taking up excess glucose and lipids to generate heat, activation of BAT can prevent obesity and metabolic abnormalities (Liu et al., 2013a; Yuan et al., 2016; O’Mara et al., 2020; Orava et al., 2011). Furthermore, BAT is a potential endocrine organ, capable of regulating metabolism in distal organs, through secreting of factors in an autocrine/paracrine manner. For instance, BAT can secret insulin, adiponectin, and leptin, which are beneficial for improve muscle mass and preventing muscle weakness.

In this review, we analyze the metabolic mechanism underlying the crosstalk between BAT and skeletal muscle. BAT also functions as a complex and highly dynamic endocrine organ, by releasing signalling molecules, including “batokines,” and bioactive lipids known as “lipokines,” that may positively or negatively sffect signaling in skeletal muscle (Villarroya et al., 2013; Villarroya et al., 2019). Consequenntly, targeting BAT could be a promising approach for treating metabolic disease like obesity and sarcopenia, opening uo a new avenue for further exploration.

## 2 The metabolic role of brown adipose tissue and skeletal muscle

BAT and skeletal muscle are both crutial for thermogenesis. While BAT was first identified as a thermogenic organ in the 1960s (Pan and Chen, 2021), it was commonly believed that BAT only existed in newborns in humans, with low levels present in adults and no apparent physiological significance. However, recent advances in positron emission computed tomography and/or X-ray tomography (18F-FDG-PET/CT) scanning, combined with molecular identification of localized tissue sampling, have shown that functional BAT does exist in adult humans, although it does decline with age. These findings have challenged the traditional view of BAT in human physiology (Cypess et al., 2009; Virtanen et al., 2009). Moreover, adipocytes in BAT contain multilocular smaller lipid roplets to storage energy, which are also rich mitochondria contains UCP-1. UCP-1 can dissipate the proton gradient of mitochondria and generate heat to maintain temperature of the core body (Pani et al., 2022).

Recent studies have shown that myocytes and brown adipocytes are both derived from mesenchymal stem cells and share similar precursor transcriptomes (Bal et al., 2016; Bal et al., 2017; Scheele and Wolfrum, 2020; Pan and Chen, 2021; Sahu et al., 2023), and both BAT and skeletal muscle use energy substrates including fat and glucose, as fuel to generate heat (Pani et al., 2022). From a metabolic perspective, the thermogenesis of BAT and skeletal muscle is in a dynamic balance. The shivering thermogenesis of skeletal muscle (e.g., skeletal muscular contraction) is responsible for heat generation in humans (Betz and Enerback, 2018), thus it is related to locomotion (Pani et al., 2022). In addition, both BAT and skeletal muscle are important sites of NST in mammals and rodents (Bal et al., 2016; Bal et al., 2017). It is assumed that the presence of active BAT might minimize the importance of muscle based on NST. Skeletal muscle was proved to be important during cold adaptation through research using birds, which lacked BAT (Bal et al., 2016). Furthermore, it is reported that physical exercise may promote fat browning via myokines, leading to thermogenesis of BAT (Rodriguez et al., 2020).

In specific, mitochondrial uncoupling is a process during which substrate oxidation can be uncoupled from ATP production, resulting in heat loss directly. This process is considered a promising target for improving energy expenditure of the whole body (Conley et al., 2007). This is primarily because of the high oxidative capacity of muscle (van den Berg et al., 2011). The mitochondrial protein UCP1 provides the primary molecular driving force for BAT thermogenic power (Virtanen et al., 2009), as a marker of brown adipocytes (Betz and Enerback, 2018). In rodents, thermogenic activity of brown adipocytes is based on the activation of β3 adrenergic receptors (β3ARs) by norepinephrine produced by axons projecting from the hypothalamus. Produced heat spreads quickly throughout the body from the well-vascularized BAT. It is worth noting that controversy does exist, with regard to whether β3AR receptors are the main effectors of BAT stimulation in humans. Riis-Vestergaard et al. (2020) demonstrated β1AR to be the prime receptor responsible for adrenergic regulation of human BAT activity. Heat production is a consequence of fatty acid oxidation that is uncoupled from ATP production by UCP1 on the inner mitochondrial membrane. Energy is lost directly in the form of heat without ATP generation. Thus, fat is consumed by uncoupling fatty acid oxidation from ATP production, generating heat (Cannon and Nedergaard, 2004). Muscle fibers express UCP2 and UCP3, which are similar to UCP1. It is speculated that these proteins can also contribute to NST as uncoupling proteins, but high-level expression of UCP2 and UCP3 cannot compensate for the loss of UCP1 in brown adipocytes in Ucp1^−/−^ mice (Golozoubova et al., 2001; Betz and Enerback, 2018). Fasting can increase levels of UCP2 and UCP3 in skeletal muscle of humans and mice, while acute cold exposure might decrease the UCP3 expression in human muscle (Millet et al., 1997; Schrauwen et al., 2002). It seems that UCP2 and UCP3 can facilitate lipid metabolism and metabolic adaptation instead of uncoupling activity. Interestingly, skeletal muscle can also promote cold-induced thermogenesis through mechanisms unrelated to uncoupling proteins and independent of shivering through calcium cycling by SERCA (Bal et al., 2012). These findings suggest that both NST in skeletal muscle and BAT play an essential role in thermogenesis and energy expenditure, with each complementing the other.

A study proposed that overexpression of UCP1 in mice might increase skeletal muscle mitochondrial uncoupling, energy expenditure of the whole body, and prevent glucose intolerance and high-fat diet induced obesity (Choi et al., 2007). Similarly, overexpression of UCP3 can prevent insulin resistance and high-fat diet induced obesity, which is likely due to non-regulated and non-physiological mitochondrial uncoupling (MacLellan et al., 2005; Choi et al., 2007). It has been have shown that BAT status and increased BAT activity in humans may improve metabolic diseases related to obesity, especially visceral adiposity (Wibmer et al., 2021). Moreover, exposing healthy adults to cold to stimulate BAT activity has been found to improve glucose metabolism and insulin sensitivity (Orava et al., 2011). These findings suggest that BAT has great potential as a therapeutic target for obesity and diabetes treatment. In conclusion, both BAT and skeletal muscle play important roles in energy homeostasis, and utilizing their thermogenesis mechanism can be a promising strategy for treating metabolic disease.

## 3 How does brown fat thermogenesis affect muscle?

### 3.1 BAT, metabolism, and skeletal muscle

**FIGURE 1:**
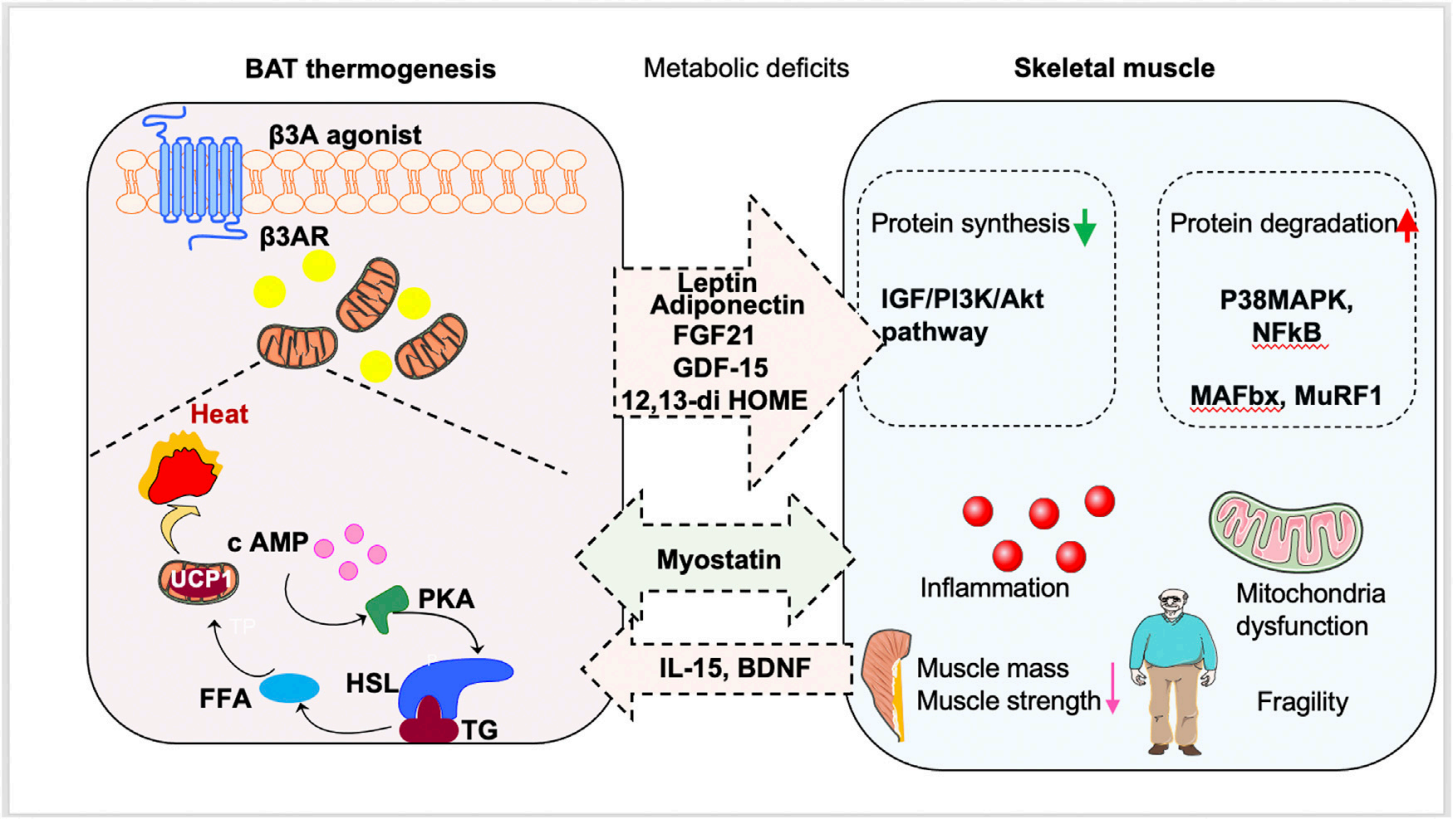
Promotion of brown fat tissue (BAT) thermogenesis as a new way to treat sarcopenia. Stimulation of BAT activity might be beneficial for sarcopenia patients by antagonizing metabolic deficits. BAT can also secrete batokines to regulate the occurrence and development of sarcopenia. Moreover, muscle-derived myokines induced by exercise can stimulate browning of white adipose tissue and delay the muscle dysfunction.

The role of muscle in energy regulation and glucose metabolism is significant. In muscle dysfunction diseases, glucose uptake is impaired, leading to metabolic dysfunction and a vicious cycle of aggravation (Eknoyan, 1999; Tournissac et al., 2021). It is possible that there is a crosstalk between BAT and skeletal muscle (Figure 1; Table 1). Additionally, stimulation of BAT activity may be beneficial for treating muscle weakness and improving glucose metabolism, which will be discussed in Section 4.

**TABLE 1:** The mechanistic crosstalk between BAT and muscle.

Hormones	Sources	Effects on muscle/BAT	Positive or negative
Insulin	BAT	Promoting glucose uptake, muscle mass↑	Positive
Adiponectin	BAT	Combining with T-cadherin to promote muscle regeneration; increase the use of fatty acid and glucose resources; protects against the degradation of muscle protein.	Positive
Leptin	BAT	Increase myonectin and myogenin transcript levels; stimulate myocyte proliferation; reduce the mRNA levels of cytokines correlated with muscle wasting.	Positive
FGF21	BAT/skeletal muscle	Increase energy expenditure, muscle strength↓; promoting WAT browing	Negative/positive
GDF15	BAT	Decrease oxidative stress; cause muscle atrophy	Negative
Myostatin	BAT/skeletal muscle	Impair exercise capacity; ↓thermogenic capacity of BAT; browning of WAT.	Negative
IL-15	skeletal muscle	Increase BAT activity and induce browning of WAT; promote the proliferation and differentiation of brown adipocyte precursor cells.	Positive
BDNF	skeletal muscle	Increase Ucp1 mRNA level and UCP1 protein expression; enhance thermogenesis.	Positive

### 3.2 Hormones regulate BAT thermogenesis and their roles in muscle function

#### 3.2.1 Positive regulators of skeletal muscle function

In clinical studies, insulin has been used to treat muscle dysfunction and improve glucose uptake (Jiménez-Osorio et al., 2016). It binds to the insulin receptor (IR) and phosphorylated insulin receptor substrate (IRS), activating the downstream phosphatidylinositol 3-kinase (PI3K)/protein kinase B (Akt) signaling pathway, which promotes glucose uptake in skeletal muscle cells (Jiménez-Osorio et al., 2016). Bouchi et al. (2017) found that patients who received insulin treatment showed higher skeletal muscle index and muscle mass in their lower extremities. A longitudinal study showed that insulin therapy preserved muscle mass but not muscle function as assessed by hand grip strength, suggesting insulin antagonism in patients with T2DM (Ferrari et al., 2020). Thus, insulin treatment may attenuate the progression of sarcopenia in patients with T2DM (Bouchi et al., 2017). BAT is known to regulate insulin signaling. In a mouse model of high-fat diet (HFD)-induced obesity, transplanted BAT significantly attenuated adipose tissue inflammation, reversed body weight and insulin resistance, and improved overall glucose tolerance. In contrast, extirpation of the inter-scapular BAT aggravated obesity, adipose tissue inflammation, and insulin resistance (Shankar et al., 2019). Moreover, insulin may regulate BAT activity. Deficiency of the insulin receptor in BAT results in a decreased thermogenic capacity for BAT and impaired glucose tolerance (Guerra et al., 2001). In diabetic mice, insulin treatment increased UCP1 expression in BAT. Maintenance of normal UCP1 requires both insulin and the sympathetic nervous system, indicating that insulin regulation of BAT thermogenic function involves sympathetic activation (Géloën and Trayhurn, 1990).

In summary, there is a link between insulin signaling and BAT activity. As such, insulin treatment may improve skeletal muscle index and potentially alleviate sarcopenia through the effects of BAT activity. However, this hypothesis requires further investigation to confirm its validity. It is important to note that while insulin has been shown to increase muscle mass in patients with T2DM, it can also increases weight gain, which must be taken into consideration (Sinha et al., 1996; Sallé et al., 2004).

Batokines are factors secreted from metabolically active BAT, and they might provide support for BAT oxidation and coordinate BAT activity with systemic metabolism (Villarroya et al., 2017; Scheele and Wolfrum, 2020).

Adiponectin is an adipokine mainly secreted from white adipose tissue (Choi et al., 2020). But BAT can also secret adiponectin, with a high degree of specificity and a multiple biological functions (Song et al., 2020). Adiponectin has garnered attention for its beneficial effects on muscles. For instance, C2C12 myoblast cells treated with globular adiponectin or a mimetic of globular adiponectin (GTDF) had significantly higher differentiation and fusion indices than vehicle-treated cells (Singh et al., 2017) via downregulation of key genes related to muscle wasting (atrogin-1 and MuRF1) (Singh et al., 2017), and GTDF could also protect rats against muscle wasting due to catabolic stimuli (China et al., 2017). Moreover, adiponectin can increase glucose uptake in muscle cells through mediation of GLUT4 (Yoon et al., 2006). Adiponectin can enhance the oxidation of fatty acids and glucose uptake by stimulating AMPK, contributing to the regulation of glucose and lipid metabolism and regulating the energy homeostasis of organisms (Kadowaki and Yamauchi, 2005; Yoon et al., 2006; Sente et al., 2016; Song et al., 2020). Thus, the therapeutic target for impaired muscle function might be adiponectin. It was shown that myoblast survival and apoptosis are inhibited by adiponectin-driven autophagy, which promotes muscle differentiation (Gamberi et al., 2016). Similarly, the evidence suggested that the levels of autophagy-related genes (e.g., LC3 and beclin-I) were decreased in skeletal muscle of adiponectin knockout mice, along with the decreased myopathic phenotype (Gamberi et al., 2016). In addition, it has been shown that the activation of adiponectin signaling may be protective against muscle wasting by combining with T-cadherin to promote muscle regeneration (Komici et al., 2021). Adiponectin’s insulin-sensitizing effect on muscleincreases the use of fatty acid and glucose resources while simultaneously promoting myogenesis. Furthermore, adiponectin protects against muscle protein degradation by upregulating the IRS-1 signaling pathway (Priego et al., 2021).

Leptin, an adipokine mainly produced by white adipose tissue and BAT, as well as the periosteum and placenta (Obradovic et al., 2021). Skeletal muscle expresses leptin receptors, which can be upregulated due to disuse atrophy (Hamrick et al., 2010). Leptin can have beneficial influences on muscle. For instance, in leptin-deficient ob/ob mice, intraperitoneal administration of leptin corrected decreased skeletal muscle atrophy and mass (Sáinz et al., 2009). Leptin administration can also increase myonectin and myogenin transcript levels, stimulate myocyte proliferation, and reduce the mRNA levels of cytokines associatedwith muscle wasting in ob/ob mice, including MuRF1 and MAFbx (Rodríguez et al., 2015). Moreover, it has been reported that leptin can increase muscle mass by reducing the expression of atrophy-associated factors, including MuRF1, myostatin, and MAFbx, in muscles, indicating that leptin is significantly related to sarcopenia severity and risk (Li et al., 2019). It has also been reported that leptin may play an important role in activating molecular pathways associated with muscle repair and regeneration. Moreover, leptin can also change the expression profile significantly in muscle-derived stem cells (Hamrick et al., 2010). Muscle regeneration may be mediated by leptin indirectly through inhibiting miR-489, and muscle satellite cells can be maintained in a quiescent state. Therefore, leptin is beneficial for improving muscle regeneration and repair (Priego et al., 2021). Leptin is also reported to have a thermogenic effect. Leptin stimulates glucose uptake by BAT, raises BAT temperature (Haque et al., 1999), and positively regulates UCP1 expression in rodent BAT (Sarmiento et al., 1997).

BAT is also a source for lipokines, which are a class of lipids that act as signaling molecules and influences systemic metabolism (Cao et al., 2008; Liu et al., 2013b; Lynes et al., 2017; Stanford et al., 2018). 12,13-dihydroxy-9Z-octadecenoic acid (12,13-diHOME) is an oxylipin, which is released from BAT following cold or exercise, has been reported to increase fatty acid uptake and oxidation in skeletal muscle (Stanford et al., 2018; Macêdo et al., 2022). Stanford et al. (2018) found that an acute bout of exercise increases plasma 12,13-diHOME levels in both humans and mice. Moreover, exercise induces the release of 12,13-diHOME from BAT, acting as an endocrine signal that stimulates fatty acid release for working skeletal muscle, and a possible role in metabolic regulation during exercise (Lynes et al., 2017). Both thermogenesis of muscle and BAT generate heat after exercise. Moreover, treatment of diet-induced obese mice with 12,13-diHOME protects against cold challenge and high-fat diet-induced obesity (Lynes et al., 2017; Shen et al., 2021). These findings suggest that 12,13-diHOME, which is induced by BAT, is involved in metabolic changes triggered by exercise.

#### 3.2.2 Negative regulators of skeletal muscle function

FGF21 is a cold-induced endocrine hormone of BAT (Lee et al., 2014) that is involved in several metabolic conditions, such as weight loss, decreased body fat, and browning of white adipose tissue, which lead to increased energy expenditure (Fisher et al., 2012). Serum FGF21 levels are significantly increased in individuals with T2DM and obesity, which can be deleterious for sarcopenic individuals with physical frailty (Zhang et al., 2008; Chen et al., 2011; Xiao et al., 2012). These contradictory observations suggest that FGF21 may be compensatory factor during disease. The role of FGF21 in muscle is similar to serum. For example, the effect of FGF21 on glucose uptake by myotubes is similar to the effect of insulin (Rosales-Soto et al., 2020), with upregulated FGF21 expression in skeletal muscle protecting against diet-induced obesity and insulin resistance (Kim et al., 2013; Pereira et al., 2017). Others found that greater FGF21 is associated with reduced handgrip strength (Conte et al., 2019), indicating that increased FGF21 acts as an adaptive regulator that counteracts muscle stress imposed by mitochondrial dysfunction. In addition, it is found that FGF21 is also an Akt-regulated myokine which can be secreted by skeletal muscle (Raschke and Eckel, 2013). As a stimulator of Akt1 signalling, resistance training exercise might improve metabolic disorders related to obesity via FGF21 production, promoting WAT browing because of endocrine effects. Thus, FGF21 is potential to be bidirectional cross-talk between BAT and skeletal muscle (Rodriguez et al., 2020).

In addition to FGF21, thermogenic BAT is also an important physiological source for GDF15 (Flicker et al., 2019). GDF15 has shown potential as a blood biomarker for human mitochondrial disorders (Fujita et al., 2020). A variety of mouse muscle models have demonstrated increased GDF15 expression in muscle, as well as increased circulating GDF15 levels during mitochondrial stress (Khan et al., 2017; Morrow et al., 2017; Ost et al., 2020; Poulsen et al., 2020). Overexpression of GDF15 in muscle reduces local muscle mass, indicating that GDF15 can lead to muscle atrophy (Conte et al., 2019; Johann et al., 2021), which is more closely related to muscle function and strength than muscle mass (Kim et al., 2020). Tang demonstrated mTORC1 activation increased the expression of GDF15 by phosphorylation of STAT3, inducing oxidative stress and catabolic changes. Blocking mTORC1 in aging mice downregulates the expression of GDF15 and STAT3’s phosphorylation in skeletal muscle, decreasing oxidative stress and muscle fiber damage and loss. These results suggest that increased GDF15 signaling may contribute to age-related muscle atrophy induced by chronically increased mTORC1 activity (Tang et al., 2019).

### 3.3 Exerkines and their roles in BAT function

#### 3.3.1 Negative regulators of BAT

Myostatin is a protein secreted by skeletal and cardiac muscles (McPherron et al., 1997) that inhibits skeletal muscle growth (McPherron et al., 1997). Myostatin can induce oxidative stress and produce ROS in skeletal muscle cells, leading to muscle wasting during sarcopenia (Sriram et al., 2011). In addition, myostatin is negatively associated with the thermogenic capacity of BAT and the browning of WAT (Braga et al., 2013; Shan et al., 2013). Kong noted that BAT controls skeletal muscle function by secreting myostatin (Kong et al., 2018). A loss of interferon regulatory factor 4 (IRF4) in BAT, which was previously recognized as a regulator of adipogenesis by the same group (Eguchi et al., 2008), resulted in increased myogenic gene expression in BAT and myostatin secretion, contributing to reduced mitochondrial function and impaired exercise capacity (Kong et al., 2018). Mice overexpressing IRF4 in BAT showed reduced serum myostatin and improved running ability compared to wild-type mice (Kong et al., 2018). Furthermore, thermo-neutral temperature increases myostatin levels in murine BAT, leading to reduced exercise capacity (Kong et al., 2018). These findings suggest that myostatin plays a dual role as a negative regulator of BAT and a regulator of skeletal muscle function as a batokine produced by BAT.

#### 3.3.2 Positive regulators of BAT

IL-15 has been shown to play a critical role in the regulation of BAT metabolism and thermogenesis (Almendro et al., 2008). It has been found to increase BAT activity and induce browning of white adipose tissue (WAT) by activating the expression of brown fat-specific genes such as UCP1, PGC-1α, and PRDM16. IL-15 also promotes the proliferation and differentiation of brown adipocyte precursor cells, resulting in more mature brown adipocytes in BAT (Duan et al., 2017). These effects of IL-15 on BAT have been shown to enhance energy expenditure and improve metabolic health, making it a potential therapeutic target for obesity and related metabolic disorders.

Cold-induced BDNF has been found to play a significant role in the regulation of BAT. Specifically, in BAT, BDNF increases Ucp1 mRNA level and UCP1 protein expression (Wang et al., 2007), and enhances thermogenesis (An et al., 2015). Studies have shown that cold exposure induces the expression of BDNF in BAT, which in turn activates the sympathetic nervous system and stimulates BAT thermogenesis. This effect is mediated by the TrkB receptor, which is expressed in BAT and binds to BDNF to promote thermogenic activity. Furthermore, BDNF has been found to enhance the browning of white adipose tissue, which makes it more metabolically active and similar to BAT. Therefore, cold-induced BDNF has potential therapeutic benefits for treating metabolic disorders by promoting BAT activity and increasing energy expenditure (An et al., 2015; Zhu et al., 2019).

In summary, these results imply that batokines produced by BAT, including adiponectin and leptin, play a positive role in skeletal muscle function. On the other hand, FGF21 and GDF15 can lead to muscle atrophy directly or indirectly through metabolism process in BAT. Moreover, myostatin, IL-15 and BNDF can also be secreted by skeletal muscle to positively or negatively regulate BAT.

## 4 Targeting BAT may be a treatment method for muscle weakness

### 4.1 Pharmacological tools that stimulate thermogenesis

β3-ARs are present in adipose tissue and β3-AR agonists are linked to increased BAT activity and the formation of brown adipocytes in subcutaneous WAT (Finlin et al., 2018). Although no clinical trials have tested β3-AR agonists in sarcopenia, studies have shown that they can improve metabolic disorders such as T2DM and obesity in both animals and humans. Only a few studies have reported the effects of β3-AR agonists on muscle. For example, Kern used the 3-AR agonist, mirabegron (50 mg/day, for 12 weeks), to treat obesity in insulin-resistant humans and found an increase in type I fibers within skeletal muscle and numerous positive effects on skeletal muscle gene expression (Finlin et al., 2020). In line with clinical results, amibegron, a β3-AR agonist induces fibro-adipogenic progenitor–BAT differentiation, improving muscle quality and shoulder function in mice with rotator cuff tears (Wang et al., 2020; Wang et al., 2021). While it is possible that β3-AR agonists directly improve muscle function, it is unclear whether they are useful in treating sarcopenia. Future clinical trials should investigate the potential benefits of such drugs At various stages of sarcopenia.

Several alternative treatments have been proposed to boost BAT activity, including GLP-1R and DPP4i (Beiroa et al., 2014; Takeda et al., 2018). Ogawa found that older diabetic patients treated with DPP4 inhibitors had a reduced risk for affixed skeletal muscle loss (Bouchi et al., 2018). Additionally, treatment with GLP-1 RA (Liraglutide, 3.0 mg per day for 24 weeks) is well tolerated, aids in fat mass loss, reduces android fat mass, and raises skeletal muscle index in T2DM patients (Perna et al., 2016).

Further, transient receptor potential (TRP) channels, such as transient receptor potential melastatin 8 (TRPM8), a calcium-gated channel, can be activated by heat and cold and indirectly stimulate BAT thermogenesis. In the murine peripheral nervous system, the TRPM8 channel severs as the primary molecular transducer for cold sensation (Bautista et al., 2007). Menthol administration activates TRPM8, resulting in elevated UCP-1 levels, enhanced temperature in humans and mice, and protection against obesity. Through TRPM8-mediated PGC1 upregulation in skeletal muscles, dietary menthol improves exercise endurance and energy metabolism, thereby enhancing muscle function (Li et al., 2018).

As a whole, these results suggest that indirect stimulation of BAT thermogenesis may be an effective strategy for muscle function enhancement. Future preclinical and clinical trials are necessary to investigate both direct and indirect methods of stimulating BAT thermogenesis in sarcopenic animals and humans.

### 4.2 Other pathways to regulate muscle atrophy through BAT

The adenosine A2B receptor is the most highly expressed G_s_-coupled G protein-coupled receptor (GPCR) to both skeletal muscle and BAT (Gnad et al., 2020). A study showed that the extracellular nucleoside adenosine playings a vital role in BAT function, and that the A2B receptor could activate energy expenditure and induce the release of the “batokine” FGF21 (Gnad et al., 2014). Additionally, A2B receptor activation has been shown to reduce diet-induced obesity. Moreover, the A2B stimulation counteracted the effects of aging on both BAT and skeletal muscle (Gnad et al., 2020).

## 5 Conclusion

Increasing BAT activity may restore thermoregulation, improve metabolic parameters, and possibly antagonize key facets of muscle dysfunction. On the other hand, BAT is an endocrine organ secreteing batokines, including adiponectin, leptin, FGF21, and GDF-15, to regulate the skeletal muscle. Myostatin is the most likely link between sarcopenia and BAT. Therefore, BAT may protect against metabolic deficits that are risk factors for skeletal muscle disease by regulation of homeostatic hormones directly and indirectly. Future research should focus on the role of BAT in muscle diseases such as sarcopenia with targeted promotion of BAT thermogenesis—a possible new means.

## Abbreviations

β3AR, β3 adrenergic receptors; AdipoRs, adiponectin receptors; AMPK, adenosine monophosphate-activated protein kinase; BAT, Brown adipose tissue; DAGs, diacylglycerols; DPP4i, dipeptidyl peptidase IV inhibitor; GLP‐1RA, glucose-like peptide-1 receptor agonist; HFD, high-fat diet; IMAT, intramuscular adipose tissue; IMCLs, intramyocellular lipids; IR, Insulin receptor; IRS, insulin receptor substrate; LD, lipid droplet; MAPK, mitogen-activated protein kinase; NRF1, nuclear respiratory factor 1; PPARα, PPAR alpha; ROS, reactive oxygen species; T2DM, type 2 diabetes mellitus; TGs, triglycerides; TRP, transient receptor potential; TRPM8, transient receptor potential melastatin 8; UCP1, uncoupling protein 1.
